# A New Method of Canine CD4^+^ T Lymphocyte Differentiation Towards the Th17 Phenotype with Analysis of Properties and Mitochondrial Activity

**DOI:** 10.3390/ijms26104946

**Published:** 2025-05-21

**Authors:** Iwona Monika Szopa, Kinga Majchrzak-Kuligowska, Rafał Pingwara, Marek Kulka, Monika Taşdemir, Małgorzata Gajewska

**Affiliations:** 1 Department of Physiological Sciences, Institute of Veterinary Medicine, Warsaw University of Life Sciences, 02-776 Warsaw, Poland; kinga_majchrzak@sggw.edu.pl (K.M.-K.); rafal_pingwara@sggw.edu.pl (R.P.); malgorzata_gajewska@sggw.edu.pl (M.G.); 2 Department of Pathology and Veterinary Diagnostics, Institute of Veterinary Medicine, Warsaw University of Life Sciences, 02-776 Warsaw, Poland; marek_kulka@sggw.edu.pl; 3 Department of Immunology, Medical University of Warsaw, 02-097 Warsaw, Poland; monika.granica@wp.pl; 4 Doctoral School, Medical University of Warsaw, 02-091 Warsaw, Poland

**Keywords:** Th17 lymphocytes, T cell activation, magnetic EpoxyBeads, Wnt/β-catenin signaling pathway, memory phenotype, T cell metabolism, interleukin-17, domestic dog model

## Abstract

Th17 lymphocytes are a distinct subpopulation of T cells that are characterized by the production of interleukins IL-17, IL-21, IL-22, and IL-26, and high expression of RORγt. These cells play an important role in inflammation and autoimmune diseases. Recent studies using rodent and human models have also highlighted their promising properties as agents in cellular immunotherapy for cancer. However, much less is known about the properties of canine Th17 lymphocytes, despite the domestic dog being an important model used in comparative medicine. In this study, we developed methods of activation and differentiation of canine CD4^+^ T lymphocytes towards the Th17 phenotype. Additionally, we targeted the Wnt/β-catenin signaling pathway to modulate the efficiency of Th17 cells differentiation. CD4^+^ T cells were successfully activated with magnetic EpoxyBeads, and in combination with the appropriate programming medium, they acquired the Th17 phenotype. Furthermore, indomethacin, an inhibitor of the Wnt/β-catenin pathway, significantly increased the efficiency of differentiation, causing elevated production of IL-17 and changed T cell metabolism by promoting oxidative phosphorylation. The protocol elaborated in our study provides an efficient method of canine Th17 lymphocyte differentiation. Our findings also suggested that the modification of the Wnt/β-catenin signaling pathway could be a valuable strategy for optimizing canine Th17 cell differentiation and advancing cell-based immunotherapy.

## 1. Introduction

Th17 cells are the subset of effector T helper lymphocytes identified over a decade ago as interleukin 17 (IL-17)-producing T cells that regulate tissue inflammation [1]. This specific subset of T helper lymphocytes is determined by their ability to produce IL-17, IL-22, IL-21, IL-26, and CC chemokine ligand 20 (CCL-20) [2]. Th17 cells also express retinoic acid-related orphan receptor gamma t (RORγt) [3]. The exact role of these cells in many physiological processes remains ambiguous, but studies have shown the involvement of Th17 cells in the course of various diseases in both humans and dogs [3,4,5]. The main function of Th17 lymphocytes is to participate in host defensive mechanisms against extracellular pathogens during infections [6,7]. Moreover, the effector cytokines produced by Th17 cells are associated with the pathogenesis of human autoimmune diseases, such as multiple sclerosis [8], rheumatoid arthritis [9], inflammatory bowel disease [10], systemic lupus erythematosus [11,12], psoriasis [13], and asthma [14]. Much less is known about the role of Th17 cells in the pathogenesis of the autoimmune diseases in dogs; however, the implication of Th17 lymphocytes has been described in the development of canine atopic dermatitis [15,16], steroid responsive meningitis-arteritis [17], and meningoencephalomyelitis [18].

Interestingly, recent studies have demonstrated that subpopulation of the Th17 lymphocytes is a promising agent in adoptive cellular immunotherapy (ACT) for cancer treatment. Adoptively transferred Th17-polarized cells exhibit a superior ability to eradicate cancer in a mouse melanoma model, compared to Th1 and Th2 cells [19,20,21,22]. Additionally, Th17 cells show a therapeutic ability to mediate durable tumor immunity and protect mice from tumor rechallenge, including lung metastasis [23]. These promising characteristics are caused by their stem cell-like properties and resistance to apoptosis, allowing the Th17 lymphocytes to maintain the potent antitumor activity after long-term ex vivo expansion [23,24].

Th17 cells, similarly to Th1 and Th2 lymphocytes, require a specific cytokine milieu for their differentiation [25]. Cytokine requirements differ slightly for mouse and human Th17 lymphocytes. However, the most important cytokine known to be involved in the induction of Th17 cell differentiation in both species is TGF-β [26]. At the initial stage of polarization, mouse T cells not only require the presence of TGF-β but also IL-6. IL-21 is then required as a cytokine that enhances the expansion of mouse Th17 lymphocytes [3,27]. To induce human Th17 differentiation, a combination of TGF-β and IL-21 is sufficient, and IL-1β and IL-6 are crucial for promoting their further proliferation [28]. The final stage of lymphocyte differentiation is the maintenance of their phenotype. The stabilization of mouse and human Th17 cells is mediated by IL-23 [3,29]. Although earlier stages of differentiation do not require IL-23, this cytokine promotes further proliferation, survival and stability of Th17 cell characteristics. Therefore, IL-23 is also crucial for Th17 differentiation in long-term in vitro cultures [30].

A crucial signaling pathway in cell differentiation during both embryonic and postnatal development is the Wnt/β-catenin signaling pathway [31]. Significant efforts have been made to understand the role of the Wnt/β-catenin signaling pathway, as it is known to regulate various crucial cellular functions in both thymic T cell development and mature peripheral T cell differentiation. Research has demonstrated that the activation of this signaling pathway in peripheral T cells can suppress their differentiation into effector cells. This effect is linked to suppressed cell division and a reduced ability to acquire effector functions, indicating that Wnt/β-catenin signaling acts as a negative regulator of effector T cell differentiation [32,33]. Moreover, the Wnt/β-catenin signaling pathway has been shown to promote self-renewal and multipotency of hematopoietic stem cells [34]. Importantly, understanding the role of Wnt/β-catenin signaling in T cell differentiation has potential therapeutic implications, as modulating this pathway may enhance the production of memory T cells, which are essential for effective immune responses [35]. However, its exact role remains not fully understood in Th17 biology. Lee et al. (2012) [36] have shown that inhibition of this signaling pathway fosters human Th17 differentiation. A similar phenomenon has been observed in murine Th17 lymphocytes [37].

So far, only a few studies have focused on the polarization of canine lymphocytes towards the Th17 phenotype [38,39], although the domestic dog is a useful model for comparative medicine. Many characteristics appear to make dogs a very attractive translational model for both immunology and oncology research [40]. Numerous features of the innate and adaptive immune system are similar in dogs and humans. In addition, the main subsets of immune cells identified in dogs have comparable phenotypes to human immune cells [41]. The dog model offers several advantages over the xenograft rodent model in oncology research, including similar tumor genetics, epidemiology, biology, prognostic factors, treatment responses, and clinical outcomes [40,42,43]. Additional similarities include the natural and spontaneous occurrence of cancer in dogs, as well as the course of disease progression [42]. Canine models are increasingly being used for the investigation of many human cancers, including sinonasal carcinoma, brain cancer, B cell lymphoma, leukemia, osteosarcoma, melanoma, and invasive uroepithelial cancers [44,45].

Therefore, the aim of this study was to develop a novel Th17 cell differentiation protocol for CD4^+^ T lymphocytes isolated from the peripheral blood of companion dogs (*Canis lupus familiaris*), improved with an effective activation method based on magnetic EpoxyBeads coated with anti-canine CD3 and CD28 antibodies. In addition, we proposed to target Wnt/β-catenin pathway and investigated the potential application of its selected modulators to enhance the in vitro differentiation of canine Th17 cells.

## 2. Results

### 2.1. CD4^+^ T Cells Isolated from Canine Peripheral Blood Are Effectively Activated by Epoxylated Magnetic Beads

**Figure 1:**
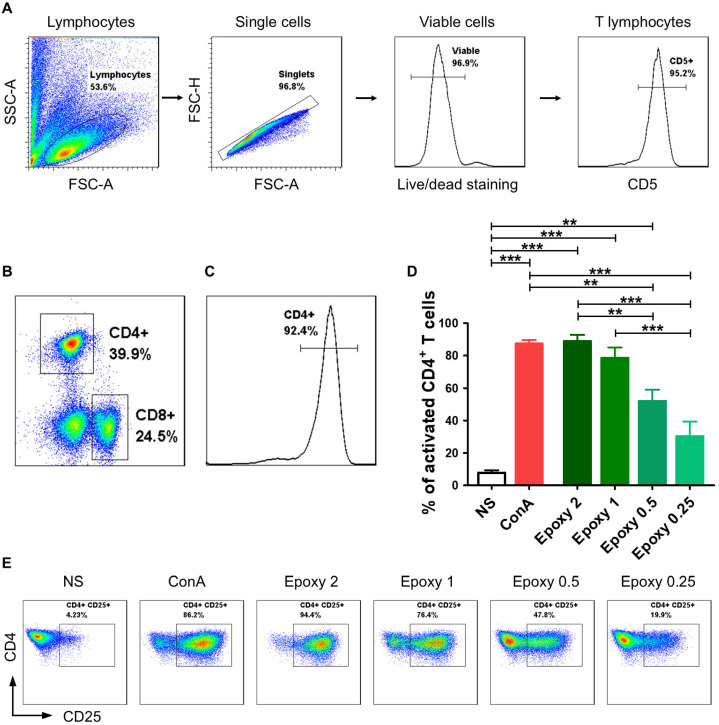
Canine CD4^+^ T lymphocytes were effectively activated using magnetic EpoxyBeads. Canine CD4^+^ T cells were isolated with the pluriBead Cell Separation Kit and analyzed by multicolor flow cytometry (FASC Aria II, Becton Dickinson). (**A**) Gating strategy for flow cytometry analysis. T lymphocytes were gated based on FSC and SSC. Only singlets and viable cells (v450 negative) were gated for further analysis. Among the analyzed cells, the vast majority consisted of CD5^+^ T cells. (**B**) Within T lymphocytes group, two subpopulations were distinguished based on CD4 and CD8α surface expression. (**C**) Isolation of CD4^+^ T cells with the pluriBead Cell Separation Kit allowed us to obtain high population purity (above 90%). (**D**) Bar graph showing mean percentage of activated canine CD4^+^ T cells 24 h post-stimulation with EpoxyBeads and ConA. Data are shown as the mean results of three separate isolations (*n* = 3), and error bars indicate SEM. Statistical analysis was performed by One-way analysis of variance (ANOVA) with Tukey’s Multiple Comparison Test (** *p* < 0.01, *** *p* < 0.001). (**E**) Representative cytograms of CD25 expression in CD4^+^ T lymphocytes after stimulation with different EpoxyBeads to CD4^+^ T cells ratios (FACS Aria II, Becton Dickinson).

The gating strategy involved distinguishing lymphocytes based on cell size and granularity (FSC vs. SSC) and excluding cell doublets (FSC-H vs. FSC-A). Only viable (Horizon v450^−^) CD5^+^ T lymphocytes were included in the further flow cytometry analyses. The vast majority of isolated lymphocytes were T cells (Figure 1A). The isolation of CD4^+^ T cells enabled us to obtain a pure population of helper T cells (Figure 1B,C).

Activation with higher concentrations of EpoxyBeads (ratios 2:1 and 1:1) allowed us to achieve a comparable effect to activation with Concanavalin A (ConA) (Figure 1D,E). The percentage of T cells expressing CD25 molecule was 88.9 ± 6.9% and 78.4 ± 11.4% (mean ± SD) at 2:1 and 1:1 EpoxyBeads to CD4^+^ T cells ratio, respectively, and 87.3 ± 3.7% when stimulated with ConA (Figure 1D,E). The two most effective ratios of EpoxyBeads to T cells (2:1 and 1:1), as well as treatment with ConA, resulted in a significantly higher expression of the CD25 activation marker (*p* < 0.001) compared to unstimulated control cells (7.8 ± 2.8%). Interestingly, lower concentrations of EpoxyBeads, applied in ratios of 0.5:1 and 0.25:1, showed weaker stimulation of activation (52.0 ± 12.2% and 30.3 ± 15.8%, respectively) (Figure 1D,E). Based on these results, the ratio 1:1 of EpoxyBeads to canine CD4^+^ T cells was selected for further experiments. Given the similar level of activation achieved by the two highest concentrations of EpoxyBeads, the choice also depended on the level of cell viability in culture. We noted that cells’ viability was higher when the 1:1 ratio was applied (Supplementary Figure S1).

### 2.2. Differentiation of Activated T Lymphocytes Towards the Th17 Phenotype Can Be Achieved with the Appropriate Programming Medium

**Figure 2:**
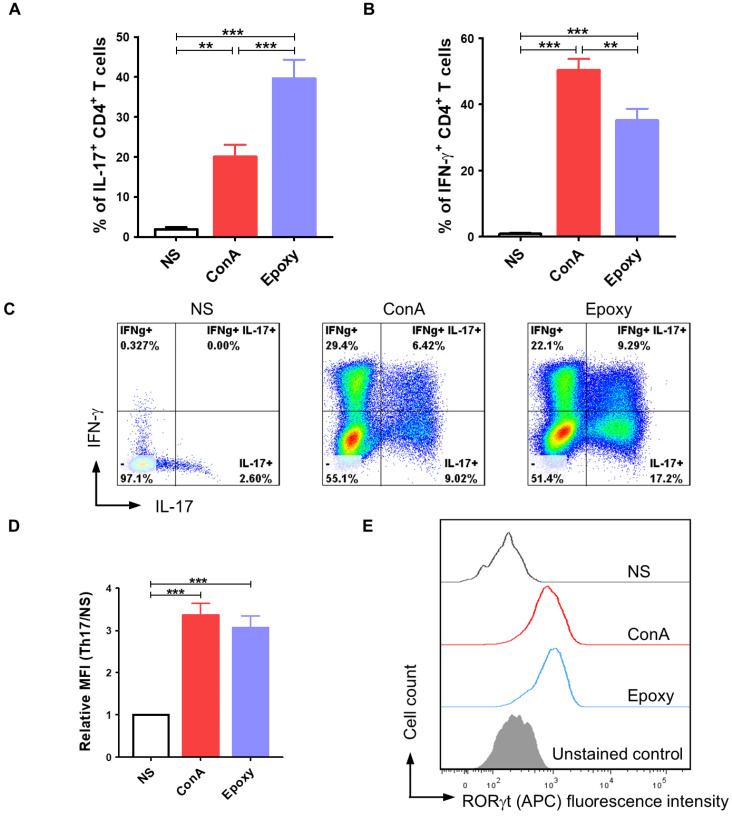
Application of a proper programming medium led to differentiation of canine CD4^+^ T lymphocytes towards the Th17 phenotype. Quantification of IL-17 (**A**) and IFN-γ (**B**) production after activation with ConA or magnetic EpoxyBeads and 10-day culture in the presence of programming medium. Data are shown as the mean results for cells isolated from at least 11 dogs, and error bars indicate SEM. (**C**) Representative flow cytometry cytograms of IL-17- and IFN-γ-producing CD4^+^ T cells. (**D**,**E**) Bar graph and representative histograms showing relative mean fluorescence intensity (MFI) of RORγt. Data are shown as the mean results for cells isolated from at least 9 dogs, and error bars indicate SEM. Statistical analysis was performed by One-way analysis of variance (ANOVA) with Tukey’s Multiple Comparison Test (** *p* < 0.01, *** *p* < 0.001).

Our next research step determined whether isolated canine CD4^+^ T cells could be successfully programmed in vitro to the Th17 phenotype. We analyzed the number of cells synthesizing IL-17, the hallmark cytokine produced by Th17 lymphocytes, and we determined the expression level of RORγt, which is the main transcription factor of Th17 cells. Additionally, the number of CD4^+^ T cells synthesizing IFN-γ was evaluated. Our data showed that application of a specific programming medium containing cytokines: IL-1β, IL-6 (as well as IL-2 and IL-23 added during long-term culture), growth factor: TGF-β and the anti-canine IL-4 antibody, resulted in T cell differentiation within 10 days of in vitro culture (Figure 2). Canine T cells activated by EpoxyBeads applied in the 1:1 ratio (EpoxyBeads to CD4^+^ T cells) responded significantly more effectively (*p* < 0.001) to the programming medium than cells treated with ConA. Among the CD4^+^ T cell population 39.6 ± 16.8% of cells showed intracellular expression of IL-17, which means that these cells actively synthesized IL-17. In the population of cells activated with ConA IL-17^+^ T lymphocytes constituted 20.0 ± 9.9% of the population. In the unstimulated cells (NS), the percentage of IL-17^+^ T lymphocytes was only 1.9 ± 1.8% (Figure 2A,C). Among the population of canine T cells activated with EpoxyBeads, 35.2 ± 12.7% of cells also expressed IFN-γ. The percentage of IFN-γ^+^ T cells in EpoxyBeads-activated T cells was significantly lower (*p* < 0.01) than in the population of ConA-activated T cells that showed 50.4 ± 11.2% of cells expressing IFN-γ. In the population of unstimulated cells, the percentage of IFN-γ^+^ T cells was significantly lower (0.9 ± 0.85%) than in T cells stimulated with EpoxyBeads or ConA (*p* < 0.001) (Figure 2B,C). In addition, the expression of the RORγt transcription factor was assessed by measuring the mean fluorescence intensity (MFI) in the analyzed canine T cell population. Compared to unstimulated controls, both ConA-activated and EpoxyBeads-activated CD4^+^ T cells showed significantly higher expression of RORγt (MFI = 3.4 ± 0.8 and 3.1 ± 0.9, respectively) (Figure 2D,E).

### 2.3. The Application of Wnt/β-Catenin Signaling Pathway Modulators Affects the Efficiency of CD4^+^ T Lymphocyte Programming Towards the Th17 Phenotype

**Figure 3:**
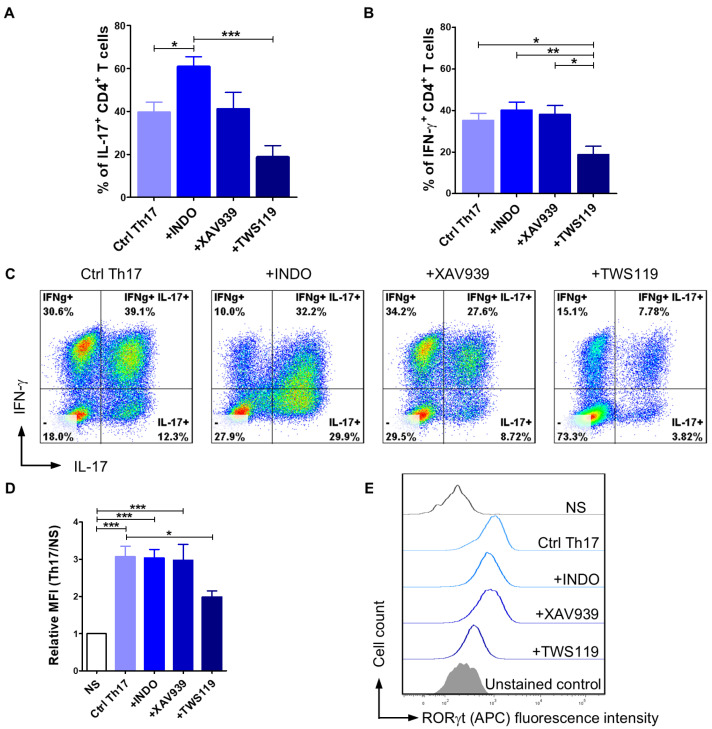
The effect of Wnt/β-catenin signaling pathway modulators on the efficiency of CD4^+^ T lymphocyte programming towards the Th17 phenotype. Quantification (**A**,**B**) and representative cytograms (**C**) of IL-17 and IFN-γ production after activation with magnetic EpoxyBeads and 10-day culture in the presence of programming medium (Ctrl Th17) and selected modifying factors of the Wnt/β-catenin signaling pathway (Indo, XAV939, TWS119). Data are shown as the mean results for cells isolated from at least six dogs, and error bars indicate SEM. (**D**,**E**) Bar graph and representative histograms showing relative mean fluorescence intensity (MFI) of RORγt. Data are shown as the mean results for cells isolated from at least 11 dogs, and error bars indicate SEM. Statistical analysis was performed by One-way analysis of variance (ANOVA) with Tukey’s Multiple Comparison Test (* *p* < 0.05, ** *p* < 0.01, *** *p* < 0.001).

To broadly assess how the modulation of the Wnt/β-catenin signaling pathway impacts differentiation towards the Th17 phenotype, we evaluated IL-17 and IFN-γ production as well as RORγt expression in CD4^+^ T cells cultured in the programming medium supplemented with indomethacin, XAV939 (Wnt/β-catenin pathway inhibitors), or TWS119 (Wnt/β-catenin pathway activator). Considering the results from previous experiments, showing that activation with EpoxyBeads promotes Th17 polarization, we designed subsequent experiments using only this stimulation method. As expected, the addition of indomethacin to the programming medium resulted in a significant increase (*p* < 0.05) in the number of IL-17^+^ T lymphocytes (61.0 ± 13.6%) when compared to control cells (Ctrl Th17) (39.6 ± 16.8%) that comprised CD4^+^ T cells activated and cultured in programming medium with no signaling pathway modulator (Figure 3A,C). Conversely, in the presence of TWS119 the percentage of IL-17^+^ T cells significantly decreased (18.7 ± 13.2%) (*p* < 0.001) compared to the indomethacin-treated cells and was lower than in control cells (Ctrl Th17) (Figure 3A,C). XAV939 had no impact on Th17 cells polarization (41.1 ± 18.9%) (Figure 3A,C). Our data indicated that neither indomethacin nor XAV939 affected IFN-γ production, as the percentage of IFN-γ^+^ T cells was similar in indomethacin-treated, XAV939-treated, and control cells (40.1 ± 13.9%, 38.0 ± 10.8%, and 35.2 ± 12.7%, respectively) (Figure 3B,C). However, the addition of TWS119 significantly reduced the number of cells synthesizing IFN-γ in the population of canine CD4^+^ T cells (18.7 ± 10.0%) (Figure 3B,C). To investigate the effect of factors modifying the Wnt/β-catenin signaling pathway on the expression of the RORγt transcription factor, we assessed the relative mean fluorescence intensity (MFI) (Figure 3D,E). Control Th17 lymphocytes and T cells treated with indomethacin or XAV939 showed a significantly higher expression of RORγt when compared to unstimulated cells (MFI = 3.1 ± 0.9, 3.0 ± 0.8 and 3.0 ± 1.0, respectively). In contrast, the addition of TWS119 to the programming medium resulted in a statistically significant decrease (*p* < 0.05) in the expression of this transcription factor when compared to Ctrl Th17 cells (MFI = 2.0 ± 0.4) (Figure 3D,E).

### 2.4. Th17 Polarization Promotes the Acquiring of an Effector-Memory T Cell Phenotype

**Figure 4:**
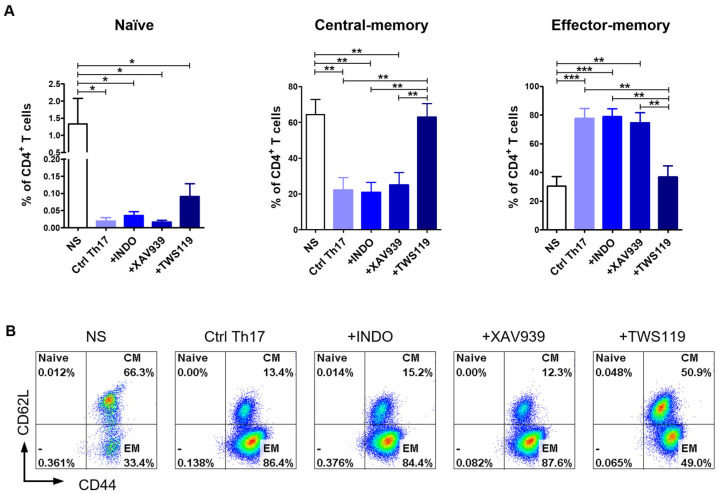
Differentiation towards the Th17 phenotype resulted in changes in the memory T cell phenotype. (**A**) Bar graph showing percentage of naïve T cells (T_Naïve_, CD44^low^CD62L^high^); central-memory T cells (T_CM_, CD44^high^CD62L^high^) and effector-memory T cells (T_EM_, CD44^high^CD62L^low^) upon 10-day differentiation of CD4^+^ T lymphocytes using selected inhibitors and an activator of the Wnt/β-catenin signaling pathway (Indo, XAV939, TWS119). Data are shown as the mean results for cells isolated from six dogs (*n* = 6), and error bars indicate SEM. Statistical analysis was performed by One-way analysis of variance (ANOVA) with Tukey’s Multiple Comparison Test (* *p* < 0.05, ** *p* < 0.01, *** *p* < 0.001). (**B**) Representative flow cytometry cytograms of different memory T cells populations under the tested conditions.

To determine the memory profile of canine CD4^+^ T cells, we defined the CD44/CD62L expression pattern. Previous studies by Rothe et al. (2017) [46] demonstrated that based on the expression of these two molecules, it is possible to distinguish three subpopulations of T lymphocytes: naïve T cells (T_Naïve_, CD44^low^CD62L^high^), central-memory T cells (T_CM_, CD44^high^CD62L^high^), and effector-memory T cells (T_EM_, CD44^high^CD62L^low^). Our flow cytometry analysis revealed that more than 75% of Ctrl Th17 cells acquired the effector-memory phenotype (T_EM_, CD44^high^CD62L^low^) (77.6 ± 16.9%) (Figure 4A,B). The stimulation of cells with indomethacin (78.8 ± 13.4%) or XAV939 (74.6 ± 16.9%) provided similar results to the control conditions (Ctrl Th17). The average percentage of central-memory T cells (T_CM_, CD44^high^CD62L^high^) in the above conditions (Ctrl Th17, indomethacin-treated, XAV939-treated) did not exceed 25% (Figure 4A,B). In contrast, TWS119 treatment caused a significant increase in the percentage of the central-memory T cell subpopulation (T_CM_, CD44^high^CD62L^high^) (62.8 ± 18.8%), with a concomitant decrease in the percentage of the effector-memory T cells (T_EM_, CD44^high^CD62L^low^) (36.9 ± 19.0%) (Figure 4A,B).

### 2.5. Differentiation Towards the Th17 Phenotype Involves Changes in Mitochondrial Activity

**Figure 5:**
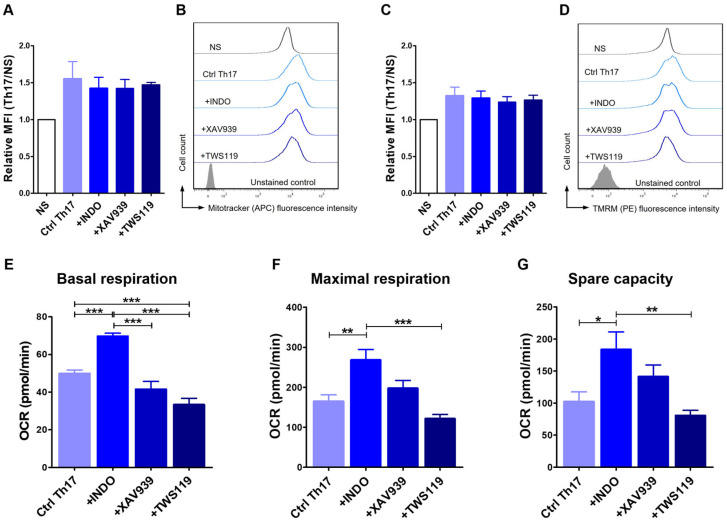
Differentiation towards the Th17 phenotype involved changes in mitochondrial activity. Relative mean fluorescence intensity (MFI) and representative histograms of MitoTracker (**A**,**B**) and tetramethylrhodamine methyl ester - TMRM (**C**,**D**) staining (FACS Aria II, Becton Dickinson) after 24 h culture in programming medium. The bar graphs show the mean results for cells isolated from 3 dogs (*n* = 3), and error bars indicate SEM. Bar graphs showing oxygen consumption rate (OCR) in terms of basal respiration (**E**), maximal respiration (**F**), and spare respiratory capacity (**G**) of CD4^+^ T lymphocytes after 72 h differentiation with programming medium and administration of selected modifying factors of the Wnt/β-catenin signaling pathway (Indo, XAV939, TWS119) (Seahorse XF HS Mini, Agilent Technologies). The bar graphs show the mean results for cells isolated from at least six dogs, and error bars indicate SEM. Statistical analysis was performed by One-way analysis of variance (ANOVA) with Tukey’s Multiple Comparison Test (* *p* < 0.05, ** *p* < 0.01, *** *p* < 0.001).

Considering that T cell metabolism changes depending on their differentiation and memory phenotype status, we examined how the differentiation of T lymphocytes towards the Th17 phenotype alters the mass and activity of their mitochondria. For this purpose, we evaluated the relative mean fluorescence intensity (MFI) after MitoTracker and TMRM staining of the mitochondria. Our study showed no statistically significant differences in mitochondrial content (MitoTracker staining) (Figure 5A,B) and mitochondrial potential (TMRM staining) (Figure 5C,D). In both cases, the average fluorescence intensity was only slightly higher in Th17-polarized cells when compared to unstimulated cells. No changes were observed between the control cells (Ctrl Th17) (1.6 ± 0.4; 1.3 ± 0.2) and cells treated with indomethacin (1.4 ± 0.3; 1.3 ± 0.2), XAV939 (1.4 ± 0.2; 1.2 ± 0.1) or TWS119 (1.5 ± 0.1; 1.3 ± 0.1) (MFI ± SD for MitoTracker and TMRM staining, respectively).

To further analyze the metabolism of Th17 lymphocytes, we performed an analysis of key parameters of mitochondrial function by directly measuring the oxygen consumption rate (OCR) using The Agilent Seahorse XF Cell Mito Stress Test. We considered three crucial characteristics, such as basal respiration, maximal respiration, and spare capacity. At the basal level, cells treated with indomethacin showed significantly higher OCR (69.7 ± 4.0 pmol/min) when compared to control (49.9 ± 6.3 pmol/min), XAV939-treated (41,58 ± 10.1 pmol/min) or TWS119-treated cells (33.7 ± 8.2 pmol/min) (Figure 5E). The latter had a statistically significantly lower OCR compared to the control cells (*p* < 0.001). However, no differences were observed between cells treated with XAV939 and control (Figure 5E). Th17 cells programmed with indomethacin were also characterized by high levels of OCR in the context of maximal respiration (268.1 ± 63.7 pmol/min). Significant differences were found when compared to control cells (164.8 ± 56.1 pmol/min), and cells treated with TWS119 (121.8 ± 25.4 pmol/min) (Figure 5F). The last evaluated parameter was spare respiratory capacity, defined as the difference between maximal and basal respiration. Spare respiratory capacity is a measure of a cell ability to respond to increased energy demands. This parameter also confirmed the increased OCR of indomethacin-treated T cells (183.8 ± 67.0 pmol/min), which were statistically significant when compared to control (102.4 ± 52.6 pmol/min) and TWS119-treated (80.7 ± 19.5 pmol/min) cells (Figure 5G).

## 3. Discussions

The domestic dog model is becoming more appreciated in comparative medicine research. Dog models of cancer are particularly valuable, as canine cancers more closely reflect human tumors biology, genetics, histology, and response to treatment [40,44]. The examination of dogs with cancer can provide information that brings a broad perspective to comparative biology. Although inbred laboratory rodents are used in standard clinical trials, the incorporation of different breeds of dogs in such studies should be regarded as an added advantage, as it also takes into account genetic variation [43]. However, there are numerous limitations regarding the procedures used for the isolation, culture, and differentiation of cells obtained from dogs [44]. Therefore, the aim of our study was to develop a protocol for effective activation and differentiation towards the Th17 phenotype of CD4^+^ T lymphocytes isolated from the peripheral blood of pet dogs. We also assessed the memory phenotype and mitochondrial activity of canine CD4^+^ T cells.

Bead-based activation has been described as accessible and effective in protocols involving both human and canine T lymphocytes. In our previous studies, we developed the method for canine lymphocyte activation using nano-sized magnetic beads coated with anti-canine CD3/CD28 antibodies. We revealed that this is an effective method to promote T lymphocyte proliferation and function, and that the higher cell culture temperature (38.5 °C) promotes the activation process [47]. However, this method did not provide satisfactory results for the activation of the isolated CD4^+^ T cell population. Hence, in the present study, we aimed to improve the activation method using epoxylated magnetic beads. These beads are much larger in diameter (4.5 µm) and offer the possibility of removing them from the cell culture once the signal for activation has been induced. Mason and colleagues also applied a method involving epoxylated magnetic beads (termed Dynabeads in their studies) [48,49,50]. They assessed the effects of activation with the plant mitogenic lectins (ConA), human immortalized erythroleukemic K562 cells (artificial antigen presenting cells, APCs) and beads-based stimulation on canine T cells. Stimulation with APCs induced the greatest T cell division and cell expansion among all activation methods used. The efficiency of activation with magnetic Dynabeads showed a wide variability depending on the donor, being effective in some canine samples and failing in others [48]. However, another study by Panjwani et al. (2020) [49] demonstrated an important advantage of activation using magnetic Dynabeads coated with anti-canine CD3 and CD28 antibodies. Lymphocytes activated with this method exhibited a higher efficiency of lentiviral transduction, highlighting the utility of this activation method in developing CAR T cell therapy. Moreover, CAR T cells activated with this approach exhibit potent anti-tumor efficacy after administration into canine patients with spontaneous diffuse large B cell lymphoma [49]. In our present study, we optimized the concentration of magnetic EpoxyBeads to ensure the most effective canine CD4^+^ T lymphocyte activation. We described higher ratios of EpoxyBeads to T cells as more effective. This agrees with the study by Panjwani et al. (2016) [48], in which a 3:1 ratio of Dynabeads to T cells was used. However, we additionally showed that the highest used concentration (2:1 EpoxyBeads to CD4^+^ T cells) simultaneously caused a decrease in T cell viability. This prompted us to use a 1:1 ratio in our experiments, as it was not significantly different from 2:1 ratio. Also, the published protocols for the clinical-scale generation of canine CAR T product from canine peripheral mononuclear cells acknowledge the possibility of successful lymphocyte activation using such a Dynabeads to T cells ratio [50].

Next, we investigated the in vitro induction of the Th17 phenotype in canine CD4^+^ T cells. Our studies showed that activated canine CD4^+^ T lymphocytes can be differentiated in the presence of the appropriate programming medium. It comprised IL-1β, IL-6, TGF-β, and the anti-canine IL-4 antibody. During long-term culture, IL-2 and IL-23 were also added. The use of these factors promoted differentiation towards the Th17 phenotype expressed by a significantly increased percentage of IL-17 producing CD4^+^ T cells. These findings are in line with previously published studies focused on the differentiation of canine Th17 cells [38,39]. Ritt et al. (2015) [39] investigated the effect of pro-inflammatory cytokines (IL-1β, IL-6, TGF-β) on production of IL-17 by canine CD4^+^ and CD8^+^ T cells. They observed a modest increase in the percentage of IL-17-producing cells among both helper and cytotoxic T lymphocytes. Results varied across the four investigated donors, achieving the highest value of 12.4% (CD4^+^IL-17^+^) and 17.6% (CD8^+^IL-17^+^). The results of another research group indicated that the use of a programming medium containing IL-1β, IL-6, TGF-β, and the anti-canine IL-4 antibody yielded almost 30% of cells producing IL-17 [38]. In both mentioned studies, the method of T lymphocyte activation was the administration of ConA [38,39]. However, our study showed that the application of magnetic EpoxyBeads for canine CD4^+^ T cell activation further enhanced the differentiation process. The percentage of canine CD4^+^IL-17^+^ T lymphocytes was significantly higher for EpoxyBeads-treated than for ConA-treated cells. Additionally, we showed that this activation method promoted the Th17 phenotype due to a significant decrease in IFN-γ production, when compared to T cells activated with ConA. Therefore, it is not only the optimal composition of the programming medium that influences the efficiency of differentiation, but also the appropriate method of T lymphocyte activation. Another difference between the method we developed and the previously published studies was the administration of IL-2 and IL-23 in the long-term culture of canine Th17 lymphocytes. IL-2 was added as a factor that promotes T lymphocyte proliferation, whereas IL-23 provided the stabilization of Th17 lymphocytes properties and their survival. A study on mouse lymphocytes demonstrated that the addition of IL-23 to in vitro cultures maintained the ability of these cells to produce IL-17. The removal of IL-23 supplementation resulted in diminished IL-17 production as well as down-regulation of Th17-related gene expression (including *Il17f*, *Il23r*, *Il22*, and *Rorc*) [30]. However, it is important to note that our research encountered several challenging stages. Th17 cells exhibit significant context-dependent plasticity, allowing them to acquire functional characteristics of Th1 cells, such as increased production of IFN-γ [51]. This phenomenon has been observed both in vivo and in vitro. For this reason, protocols for differentiating human and mouse Th17 cells include the use of an anti-IFN-γ antibody to prevent Th17 into Th1-like transformation. Therefore, we also tested the use of the anti-canine IFN-γ antibody during the optimization of the programming medium composition. We observed that the addition of anti-canine IFN-γ antibody to the programming medium did not reduce the production of IFN-γ by canine T cells. Based on this observation, we decided not to include the anti-IFN-γ antibody in the final programming medium composition. Additionally, due to the possible presence of Th1-like cells, we attempted to determine the percentage of Th1 cells in the obtained cell population by assessing the expression level of the T-box transcription factor T-bet, which is characteristic of Th1 cells. However, we were unable to do so because of the limited availability of canine-specific antibodies. In contrast to humans and mice, studies in dogs are hindered by a more restricted repertoire of commercially available reagents, a concern also highlighted by other researchers [52]. Nevertheless, it is worth highlighting that we obtained the highest percentage of canine Th17 cells in in vitro culture compared to results from other studies published so far. In addition, while developing the canine Th17 cell differentiation protocol, we also considered its potential for future use in generating lymphocytes for adoptive cell transfer (ACT). Remarkable features of Th17 cells in the context of ACT, such as higher in vivo survival, self-renewal capacity and less senescence, are considered to be associated with the superior plasticity of this subset compared to polarized Th1 cells, which have less plasticity and higher phenotypic stability [51]. These features seems to be necessary for antitumor activity of Th17 cells in ACT therapy, and the ability to produce IFN-γ, may even enhance their anti-tumor properties, as previously demonstrated in a mouse model [37].

Considering the studies on human [36] and mice [37] T lymphocytes, we hypothesized that modification of the Wnt/β-catenin signaling pathway may promote the differentiation of canine Th17 cells in vitro. The Wnt/β-catenin signaling pathway was previously found to promote self-renewal and multipotency of haematopoietic stem cells and to regulate the development of thymocytes at various stages [53,54,55,56]. Furthermore, the Wnt/β-catenin signaling cascade is considered to play a complex role, not only in regulating T cell development, but also in the differentiation of mature peripheral T cells [33,57]. In our study, we examined the effect of three factors modifying the signaling pathway: indomethacin, XAV939, and TWS119. Indomethacin, although does not belong to the group of specific Wnt/β-catenin pathway inhibitors, causes a downregulation of β-catenin transcription, as well as an induction of protein degradation [58]. XAV939 is a small molecule inhibitor of the Wnt/β-catenin pathway, that functions through binding to tankyrase and stabilizing the Axin protein, thereby preventing its degradation. Axin is a key component of the β-catenin destruction complex. It promotes phosphorylation, enhances degradation of β-catenin, and reduces β-catenin nuclear accumulation, leading to reduced transcriptional activation of Wnt target genes [59,60]. TWS119 inhibits glycogen synthase kinase-3β (GSK-3β) and thus activates Wnt/β-catenin pathway, causing the accumulation and nuclear translocation of β-catenin [61]. Our study revealed that the administration of indomethacin resulted in a significant increase in the number of canine CD4^+^ T lymphocytes producing IL-17 in comparison to control cells. Previous studies showed a similar effect on mouse T lymphocytes [37]. However, studies performed by Kol and colleagues (2016) [38] on canine cells did not show similar results. The addition of indomethacin did not significantly increase IL-17 production. Importantly, the concentration of the inhibitor (10 µM) used by Kol et al. (2016) [38] was six-fold lower when compared to the protocol we developed (60 µM). Despite the use of two different inhibitors in our study, only indomethacin promoted Th17 differentiation, while XAV939 had no significant effect on the examined properties of T cells. The discrepancies in the effect of the two inhibitors used, XAV939 and indomethacin, may result from differences in their mode of action on β-catenin. Indomethacin is a well-known nonsteroidal anti-inflammatory drug (NSAID) that nonselectively inhibits cyclooxygenase enzymes (COX-1 and COX-2) [62]. Importantly, reports indicated an inhibitory effect of prostaglandins (PGE2) on the differentiation of human and murine Th17 lymphocytes [63]. However, studies conducted on canine T cells by Kol and colleagues (2016) [38] clearly demonstrated the absence of such an effect. Therefore, the impact of indomethacin in the context of prostaglandin-mediated effects on Th17 lymphocyte differentiation was not addressed within the framework of this study. Additionally, indomethacin acts as a non-specific modulator of several signaling pathways. It has been described as a modulator of NF-κB [64,65], PI3K/Akt [66], as well as a Wnt/β-catenin signaling pathway [67,68,69], making it an interesting bioactive molecule, that can be used in various studies on modifications of cell signaling pathways. The application of indomethacin in our research significantly enhanced the differentiation of canine CD4^+^ T cells towards the Th17 phenotype, increasing IL-17 production and the expression of the transcription factor RORγt. The opposite effect to that observed following indomethacin administration was observed after the addition of the Wnt/β-catenin signaling pathway activator, TWS119, that significantly impaired the differentiation of canine CD4^+^ T cells. The percentage of IL-17^+^, as well as RORγt^+^ T cells was clearly decreased compared to control cells, although the results were not statistically significant due to differences among blood donors. However, the results significantly decreased compared to the indomethacin-treated cells. In addition, the percentage of IFN-γ^+^ T cells was significantly lower in comparison to all other tested conditions. Similar research using canine T cells has not been published so far, but studies on mouse models confirm that applications of TWS119 contribute to the arrest of T lymphocytes differentiation. Gattinoni et al. (2009) [70] showed that the addition of TWS119 to pmel-1 transgenic TCR mouse CD8^+^ T cells led to inhibition of their differentiation and function. Our observations agree also with studies on human T lymphocytes conducted by Muralidharan et al. (2011) [33]. The group reported general differentiation inhibition of T cells derived from peripheral blood as well as umbilical cord blood. One of the characteristic features was a decrease in IFN-γ production after TWS119 administration (17% ± 11%), compared to control cells (41% ± 16%). The described percentage of IFN-γ^+^ T cells appears very similar to that obtained in our study. Muralidharan et al. (2011) [33] also assessed the expression of transcription factors and cytokines associated with several T lymphocyte subpopulations, including Th17 cells. Similarly to our results, TWS119-treated cells showed reduced expression of RORγt and IL-17. Results regarding other parameters suggest that TWS119 does not affect a specific subpopulation of CD4^+^ T cells (Th1, Th2, Th17 or Treg), but rather has similar inhibitory effects on all subsets [33]. Overall, the administration of indomethacin enhanced canine Th17 lymphocyte differentiation. Although we did not observe a definitive action of XAV939 in this context, the clear impact of TWS119, diminishing canine Th17 cell differentiation via Wnt/β-catenin signaling pathway activation, supports the hypothesis that the Wnt/β-catenin signaling pathway is involved in this process.

In the next step of our study, we categorized the canine CD4^+^ T cells into different subsets based on their migration patterns, which provide insights into their functional roles. Previous studies conducted by Rothe et al. [46] indicated the possibility of using CD44/CD62L surface expression to distinguish the following canine T cell subpopulations: naïve T cells (T_Naïve_, CD44^low^CD62L^high^); central-memory T cells (T_CM_, CD44^high^CD62L^high^), and effector-memory T cells (T_EM_, CD44^high^CD62L^low^). CD62L (L-selectin) is a marker present on naïve T cells and further allows to differentiate central-memory (CD62L+) fromeffector-memory (CD62L^−^) T cells, as it is shed from the cell membrane upon T cell activation. The regulation of CD62L is crucial for controlling the trafficking of T lymphocytes to and from peripheral lymph nodes [71]. CD44, in turn, is upregulated following the activation of naïve T lymphocytes in response to antigen stimulation and remains elevated on the surface of memory T cells, which mediate the secondary immune response [72]. Our study showed a high percentage of T_EM_ lymphocytes (above 75%) upon differentiation of cells in the programming medium alone, and with the addition of Wnt/β-catenin signaling pathway inhibitors (indomethacin and XAV939). These results indicate that canine CD4^+^ T cells provided with an appropriate activation stimulant and differentiated in the programming medium acquire an effector phenotype. It may be characterized by a preferential migration to peripheral tissues and rapid mediation of effector functions [73]. In addition, this process was not significantly affected by the addition of indomethacin or XAV939. So far, no studies have characterized how modifications of the Wnt/β-catenin signaling pathway affect the memory phenotype of canine T lymphocytes. However, a similar pattern was described for mouse and human T lymphocytes, where an increase in CD44 expression was observed during T cell differentiation, with a simultaneous gradual loss of CD62L expression [74]. In contrast, the application of TWS119, an activator of the Wnt/β-catenin signaling pathway, led to a markedly increased percentage of T_CM_ cell subpopulation, accompanied by a decrease in the percentage of T_EM_ cells. Several research groups described the inhibitory effect of TWS119 on differentiation of human and mice T cells [33,70]. Gattinoni et al. (2009) [70] demonstrated that TWS119 arrested differentiation of CD8^+^ T cells and negatively regulated differentiation of effector T cells. Administration of TWS119 increased the frequency of T cells that retained CD62L expression, which is typical of naïve T cells and T_CM_ [70]. Data published so far suggest that Wnt/β-catenin signaling pathway is a negative regulator of T cell differentiation in human and mice T lymphocytes [33,70]. Thus, additional activation of this signaling pathway promotes the arrest of T cell differentiation, which was also confirmed in our study.

Many recent studies have focused on how activity, differentiation stage and memory phenotype affect T lymphocyte metabolism [75,76,77,78]. In our study, we also assessed how differentiation towards the Th17 phenotype impacts mitochondrial mass and activity. Flow cytometry analysis revealed that mitochondrial mass content as well as mitochondrial potential were increased in all experimental conditions compared to non-stimulated cells. However, these differences did not reach statistical significance. To determine the in-depth energy utilization of canine lymphocytes programmed towards the Th17 phenotype, we used real-time measurement of the oxygen consumption rate (OCR). We observed that the addition of indomethacin led to the highest rates of canine Th17 cell stimulation and differentiation, which was reflected in the level of OCR. These T cells were characterized by the most efficient oxidative phosphorylation (OXPHOS) in terms of basal and maximum respiration rates, as well as spare respiratory capacity. In contrast, the administration of TWS119 caused a significant decrease in OCR for all tested parameters (basal and maximum respiration, spare respiratory capacity). Our data suggest that the addition of indomethacin, which among others is known to inhibit the Wnt/β-catenin signaling pathway, and thus yields a higher percentage of Th17 cells, results in a shift in metabolism in favor of OXPHOS rather than glycolysis. T lymphocytes employ different metabolic pathways depending on their differentiation and memory status [75,76,77,78]. Naïve and memory T cells use OXPHOS to survive in a quiescent state. However, exposure to antigen results in naïve T cell differentiation into effector cells and in metabolic changes, including the use of glycolysis for efficient cytokine secretion. Our results show some discrepancy with these tendencies, indicating the use of OXPHOS by canine Th17 cells, which exhibit an effector-memory phenotype. However, the current literature indicates that T_EM_ cells use both glycolysis as well as the mitochondrial machinery, allowing them to respond rapidly to secondary antigen encounter by the production of effector cytokines [75]. Additionally, recent studies focusing on Th17 cells revealed that they rapidly increase mitochondrial respiration during development. Moreover, this process appeared to be necessary for metabolic reprogramming upon T cell activation [79]. It may be associated with the stemness properties of Th17 cells and the ability of these long-lived T cells to survive and maintain an effector phenotype [80]. Importantly, it also has been shown that modifications of various signaling pathways can lead to the reprograming of T cell metabolism and changes in mitochondrial fitness [81]. So far, the role of Wnt/β-catenin signaling pathway in regulating T cell metabolism has not been studied. However, it has been shown that the overstimulation of the Wnt/β-catenin signaling pathway in glioma cells shifts their metabolism toward glycolysis [82]. This is consistent with our findings concerning TWS119-treated T cells, which showed a reduced OCR when compared to T lymphocytes cultured with indomethacin. This observation may suggest increased glycolysis in T lymphocytes with an activated Wnt/β-catenin signaling pathway. Overall, heterogeneous subsets of T cells present differences in metabolic commitment, even within classically defined populations [83].

Our study has certain limitations that should be considered. The primary limitation is the inability to determine the precise mechanism by which indomethacin promotes the differentiation of CD4^+^ T lymphocytes towards the Th17 phenotype. Despite the significant effect following the addition of indomethacin to the programming medium, we were unable to definitively elucidate the underlying mechanism responsible for this process. As indomethacin is a non-specific modulator, future research should investigate its effects in the context of various signaling pathways, not only the Wnt/β-catenin signaling pathway. Additionally, the distinct effects of prostaglandins on Th17 lymphocyte differentiation should be explored in future studies. Moreover, a negative effect of Wnt/β-catenin pathway activation via TWS119 is apparent. Reversing this mechanism through the activation of GSK-3β could enable the assessment of the impact of Wnt/β-catenin signaling inhibition. A comprehensive investigation of the biological mechanisms underlying Th17 lymphocyte differentiation was beyond the scope of this study. Nonetheless, our findings provide a solid foundation for further research.

In conclusion, despite the aforementioned limitations, this study demonstrated the method of canine Th17 lymphocyte differentiation in vitro as well as phenotypic and metabolic changes that are involved in this process. The research confirmed that canine CD4^+^ T lymphocytes isolated from the peripheral blood can be successfully activated with EpoxyBeads coated with anti-canine CD3 and CD28 antibodies, and the use of proposed programming medium effectively promotes the differentiation of these cells towards the Th17 phenotype. Additionally, we characterized these cells as effector-memory T lymphocytes. To the best of our knowledge, this is the first study that combined an activation method based on engagement of T cell receptor (CD3) and a costimulatory molecule (CD28) together with culture in the programming medium to obtain high percentage of canine Th17 cells. Moreover, in our study we investigated the potential application of Wnt//β-catenin signaling pathway modulators to further enhance the efficiency of canine Th17 lymphocyte differentiation process. The administration of indomethacin, which affects various signaling pathways, including its inhibitory role on the Wnt/β-catenin pathway activity, led to a significant increase in the number of canine CD4^+^ T cells producing IL-17. These findings suggest that indomethacin may be further exploited in the future, with reference to its stimulatory role in Th17 lymphocyte differentiation. Furthermore, by assessing mitochondrial activity, we discovered these cells were characterized by the most efficient oxidative phosphorylation. Given the promising properties of Th17 cells in adoptive cellular immunotherapy, this research not only broadens the understanding of canine Th17 lymphocyte biology but may also contribute to the development of adoptive immunotherapy in veterinary medicine.

## 4. Materials and Methods

### 4.1. Isolation of Canine Peripheral Blood CD4^+^ T Lymphocytes

Canine peripheral blood was obtained from healthy donors of Warsaw Specialist Blood Bank. All donors included in the study were vaccinated up to date against infectious diseases (Parvovirus, Distemper virus, Adenovirus-2, Parainfluenza virus and Rabies) and were regularly dewormed. The anticoagulated blood was diluted with sterile PBS in a ratio 1:2 prior administration to a density gradient medium (Lymphoprep, Stemcell Technologies, Vancouver, BC, Canada) that was used to isolate canine peripheral blood mononuclear cells (PBMCs). Density gradient centrifugation was performed in SepMate PBMC isolation tubes (Stemcell Technologies, Canada) at 800× *g*, 10 min, at room temperature (RT). Afterwards, the isolated cells were washed twice with PBS supplemented with 2mM EDTA and 2% FBS and treated with erythrocyte lysis buffer (ACK lysing buffer, Thermo Fisher Scientific, Waltham, MA, USA) for 4 min at RT. After the erythrocyte lysis procedure, the remaining cells were washed with sterile PBS and enumerated using automated cell counter (Countess II Automated Cell Counter, Thermo Fisher Scientific, Waltham, MA, USA). To determine viability of isolated PBMC, staining with 4% Trypan blue (Thermo Fisher Scientific, Waltham, MA, USA) was used. Cells were resuspended in T cell medium composed of RPMI-1640 GlutaMAX™ medium supplemented with 10% FBS, 1% sodium pyruvate, 1% nonessential amino acids, 0.1% HEPES, and 1% antibiotics: penicillin and streptomycin (all from Thermo Fisher Scientific, Waltham, MA, USA). PBMCs were cultured overnight (38.5 °C, 5% CO_2_) at a density of 2 × 10^6^ cells/mL in 6-well plates (Corning, New York, NY, USA) for monocyte/macrophage depletion via plastic adherence. The following day, CD4^+^ T cells were isolated using pluriBead Cell Separation Kit and S-pluriBeads coated with anti-canine CD4 antibodies (pluriSelect Life Science, Leipzig, Germany) according to the manufacturer’s instructions.

### 4.2. Activation and Differentiation of CD4^+^ T Cells

Our previous research demonstrated that canine T lymphocytes are effectively activated by magnetic MicroBeads [47]. In the present study, we optimized the method of canine CD4^+^ T cell activation by testing the effect of using different ratios of epoxylated magnetic beads (Dynabeads™ M-450 Epoxy, Thermo Fisher Scientific, Waltham, MA, USA), described as “EpoxyBeads”, coated with cross-linking anti-canine CD3 (clone CA17.2A12, Bio-Rad, Hercules, CA, USA) and anti-canine CD28 (clone 1C6, Functional Grade, eBioscience, Thermo Fisher Scientific, Waltham, MA, USA) antibodies in the concentration recommended by the manufacturer. For the activation assessment, CD4^+^ T cells were seeded at a density of 2 × 10^6^ cells/mL in 48-well plates (Corning, New York, NY, USA) in T cell medium and activated with EpoxyBeads. To optimize the method and ensure the best possible activation status, CD4^+^ T cells were incubated for 24 h at either 2:1, 1:1, 0.5:1 or 0.25:1 ratio of EpoxyBeads to T cells or with 5 µg/mL Concanavalin A (ConA, natural mitogen) (Thermo Fisher Scientific, Waltham, MA, USA) that was used as a positive control. Upon 24 h of stimulation using EpoxyBeads or ConA we analyzed the surface expression of activation marker—CD25 molecule (an α chain of receptor for IL-2) using flow cytometry.

**Figure 6:**
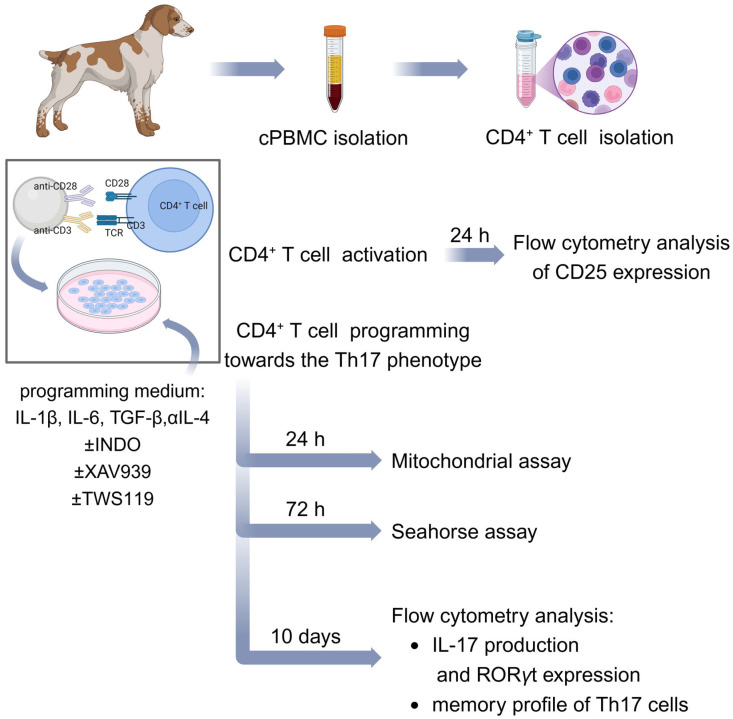
Summarized methodology of the study. Schematic representation of canine CD4^+^ T cell activation and differentiation towards the Th17 phenotype described in Section 4. Created in BioRender. Gajewska, M. (2025) https://BioRender.com/v89x776.

In order to differentiate cells towards the Th17 phenotype, lymphocytes were activated with EpoxyBeads or ConA, and a programming medium was used, composed of T cell medium (described above) with addition of recombinant canine IL-1β (10 ng/mL), recombinant canine IL-6 (5 ng/mL), recombinant human TGF-β (2 ng/mL) and anti-canine IL-4 antibody (2 ng/mL) (all from R&D Systems, Minneapolis, MN, USA). For long-term culture, EpoxyBeads were removed from the culture on day 3, and the medium was additionally supplemented with recombinant canine IL-2 (10 ng/mL) (R&D Systems, Minneapolis, MN, USA) and human recombinant IL-23 (30 ng/mL) (Gibco, Thermo Fisher Scientific, Waltham, MA, USA). To remove the EpoxyBeads, the suspension containing T cells and beads was pipetted and transferred to 2 mL Eppendorf tubes (Eppendorf, Hamburg, Germany). The tubes were then placed in a magnetic rack (Bio-Rad, Hercules, CA, USA). After 3 min, the medium containing lymphocytes was carefully transferred back to the culture plates (Corning, New York, NY, USA). In addition, in experiments evaluating the effect of Wnt/β-catenin signaling pathway modification, the programming medium was supplemented with selected signaling pathway modifying factors: indomethacin (60 µM, inhibitor of Wnt/β-catenin pathway), XAV939 (1 µM, a Tankyrase inhibitor, thereby inhibiting Wnt/β-catenin signaling) or TWS119 (2 µM, a Wnt/β-catenin pathway activator) (all from Sigma-Aldrich, Taufkirchen, Germany). Cells were cultured at 38.5 °C in a humidified incubator, 5% CO_2_ (Sanyo Electric., Ltd., Osaka, Japan), harvested on the days indicated and used for flow cytometry analysis and real-time cell metabolic analysis performed using Seahorse XFp Cell Mito Stress. The schematic workflow illustrating the main steps of the study is presented in Figure 6.

### 4.3. Flow Cytometry Analysis

CD4^+^ T cells were washed with sterile FACS buffer (PBS supplemented with 2% FBS). To block non-specific antibody binding, the cells were pretreated with Fc Receptor Binding Inhibitor Polyclonal Antibody (eBioscience™, Thermo Fisher Scientific, Waltham, MA, USA) for 20 min at 4 °C. Next, the cells were resuspended in 100 µL of FACS buffer and stained with LIVE/DEAD™ Fixable Aqua Dead Cell Stain Kit (Invitrogen, Thermo Fisher Scientific, Waltham, MA, USA). At the same time, an appropriate amount of mouse primary antibodies was added, and the cells were stained for 20 min at RT in dark. The following antibodies were used to analyze the expression level of surface molecules and activation markers: anti-canine CD4-APC (clone YKIX302.9), anti-canine CD8a-v450 (clone YCATE55.9), anti-canine CD5-PerCP-eFluor710 (clone YKIX322.3), and anti-canine CD25-FITC (clone P4A10) (all from eBioscience™, Thermo Fisher Scientific, Waltham, MA, USA). In addition, rat anti-canine CD44-Alexa Fluor 488 (clone YKIX337.8.7) and mouse anti-human CD62L-PE (clone FMC46) antibodies (Bio-Rad, Hercules, CA, USA) were used for memory phenotype staining. After incubation with antibodies, cells were washed twice with FACS buffer, centrifuged at 300× *g* for 4 min and resuspended in 200 µL of FACS buffer for analysis.

The mitochondrial assay involved staining with two dyes: Tetramethylrhodamine methyl ester (TMRM) and MitroTracker (both from Invitrogen, Thermo Fisher Scientific, Waltham, MA, USA). CD4^+^ T cells were harvested after 24 h of culture, resuspended in warm T cell medium with TMRM (250 nM) and Mitotracker (125 nM), and incubated for 30 min at 37 °C protected from light. The cells were then centrifuged and resuspended in 200 µL of warm FACS buffer for cytometric analysis.

Flow cytometry analyses were performed using a BD FACS Aria II flow cytometer (Becton Dickinson, Heidelberg, Germany). Each time, at least 50,000 events per sample were recorded. Data obtained were analyzed with FlowJo 7.6.1 software (TreeStar Inc., Ashland, OR, USA). Lymphocytes were gated according to their size and granularity using forward-scatter (FSC) and side-scatter (SSC) parameters. Dead cells and doublets were excluded from further analysis. Gating strategy is presented in Figure 1A.

### 4.4. Flow Cytometric Analysis of Cytokines and Transcription Factors Characteristic of Th17 Cells

In order to achieve differentiation of canine CD4^+^ T lymphocytes towards the Th17 phenotype, the cells were cultured for 10 days in the programming medium described in Section 2.2. Next, the cells were re-stimulated for 6 h with 15 ng/mL Phorbol-12-Myristate-13-Acetate (PMA) and 500 ng/mL Ionomycin (both from R&D systems, Minneapolis, MN) in the presence of Brefeldin A (eBioscience, Thermo Fisher Scientific, Waltham, MA, USA) added 2 h after the beginning of incubation. This step allowed stimulation of cytokine synthesis with concomitant inhibition of cytokine secretion. Subsequently, CD4 surface antigen was stained with rat anti-canine CD4-PE-Cyanine7 (clone YKIX302.9) (eBioscience, Thermo Fisher Scientific, Waltham, MA, USA), followed by an intracellular staining for cytokines and transcription factors performed using Foxp3/Transcription Factor Staining Buffer Set (eBioscience, Thermo Fisher Scientific, Waltham, MA, USA). Cells were washed and resuspended in Fixation/Permeabilization buffer for 45 min at 4 °C. Next, cells were suspended in primary antibodies diluted in permeabilization buffer and incubated for 20 min at RT. The following antibodies with confirmed canine cross-reactivity were used for intracellular staining: anti-human IL-17A-PerCP-Cyanine5.5 (clone eBio64DEC17), anti-human/mouse RORγt-APC (clone AFKJS-9) (both from eBioscience, Thermo Fisher Scientific, Waltham, MA, USA), and mouse anti-bovine IFN-γ-Alexa Fluor 488 antibody (clone CC302) (Bio-Rad, Hercules, CA, USA). After staining, cells were washed twice with FACS buffer and analyzed using BD FACS Aria II flow cytometer (Becton Dickinson, Heidelberg, Germany) with the principles described above.

### 4.5. Seahorse Assay

The oxygen consumption rate (OCR) was determined using a Seahorse XF HS Mini analyzer and the Seahorse XFp Cell Mito Stress Test Kit (Agilent Technologies, Santa Clara, CA, USA) according to the manufacturer’s instructions. CD4^+^ T cells were activated and cultured for 72 h in the presence of programming medium and factors modifying Wnt/β-catenin signaling pathway (as described above). Afterwards, cells were seeded onto Seahorse XFp 8-well plates (Agilent Technologies, Santa Clara, CA, USA) at a density of 0.5 × 10^6^ cells per well/180 µL in Seahorse XF RPMI Medium supplemented with 1 mM pyruvate, 2 mM L-glutamine and 10 mM glucose (all from Agilent Technologies, Santa Clara, CA, USA). Cell culture plates were incubated for 1 h at 37 °C in a non-CO_2_ incubator prior to analysis. The corresponding Seahorse medium was used to prepare the solutions of all compounds using in the assay. The following volumes of each compound were pre-loaded into the respective ports of the cartridge: Port A: oligomycin, 20 µL, final concentration: 1.5 µM; Port B: FCCP, 22 µL, final concentration: 1.5 µM; Port C: a mix of rotenone and antimycin A, 25 µL, final concentration: 0.5 µM. All measurements were performed in duplicate. The data obtained were analyzed with Agilent Seahorse Analytics software available online (https://seahorseanalytics.agilent.com; accessed on 12 November 2024) (Agilent Technologies, Santa Clara, CA, USA).

### 4.6. Statistical Analysis

Statistical analysis of data was performed using GraphPad PrismTM 5.0 (GraphPad Software Inc., San Diego, CA, USA). For comparisons between multiple groups, a One-way ANOVA followed by Tukey’s multiple comparisons post hoc test was performed (as indicated in the figure legends). Symbols indicate significant differences between the specified groups, as follows: * *p* < 0.05; ** *p* < 0.01; *** *p* < 0.001.

## Data Availability

The original contributions presented in this study are included in the article and Supplementary Materials. Further inquiries can be directed to the corresponding author.

## Supplementary Materials

The following supporting information can be downloaded at: https://www.mdpi.com/article/10.3390/ijms26104946/s1.
