# Supplementary material for: A New Method of Canine CD4+ T Lymphocyte Differentiation Towards the Th17 Phenotype with Analysis of Properties and Mitochondrial Activity
Source: Int J Mol Sci. 2025 May 21;26(10):4946. doi: 10.3390/ijms26104946 (PMC12112516; doi:10.3390/ijms26104946)
Supplement: Supplementary file 1 [file ijms-26-04946-s001.zip › ijms-3568861-supplementary.pdf]

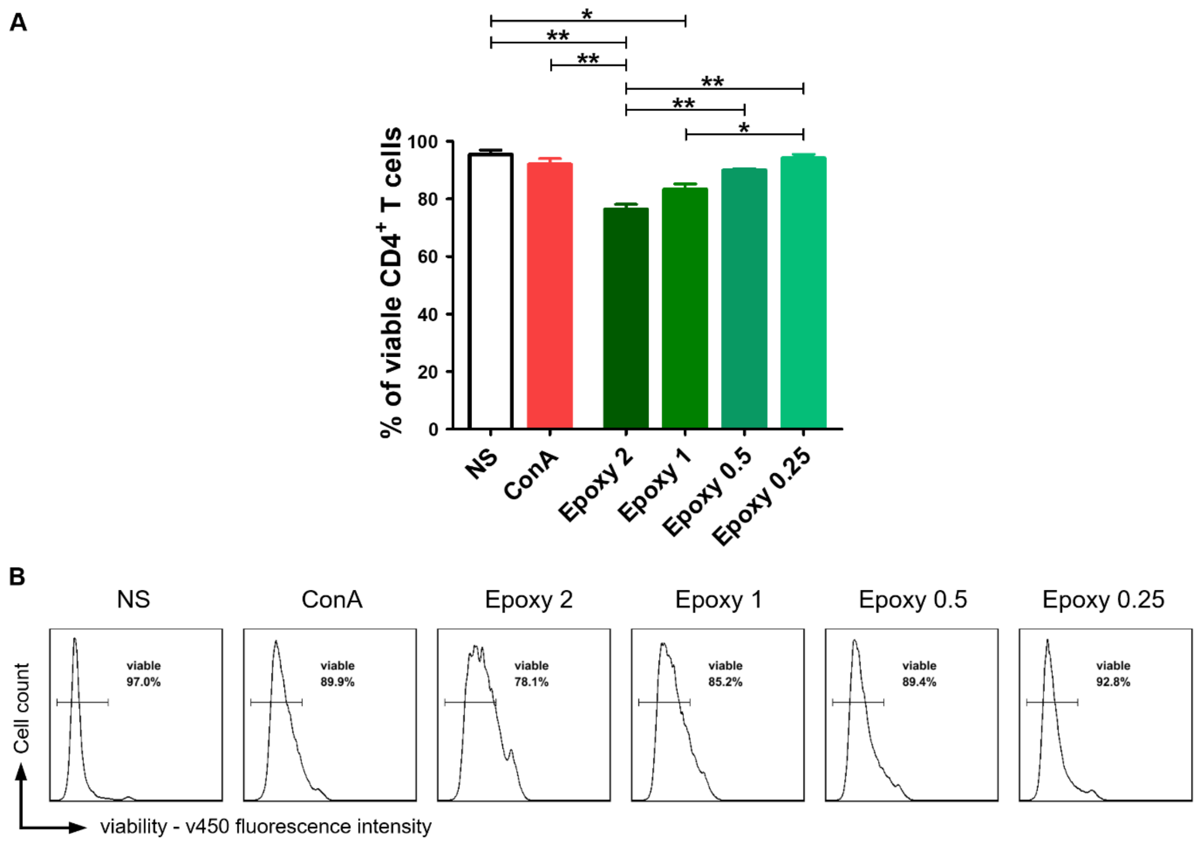

Supplementary Figure S1

The amount of EpoxyBeads applied to activate CD4+ T cells affected cell viability. (**A**) Bar graph showing mean percentage viability of activated canine CD4+ T cells 24 h post-stimulation with ConA and different ratios of EpoxyBeads. Data are shown as the mean of 3 dogs (n=3), and error bars indicate SEM. Statistical analysis was performed by One-way analysis of variance (ANOVA) with Tukey's Multiple Comparison Test (*$p < 0.05$, **$p < 0.01$). (**B**) Representative histograms of cell viability after stimulation with different EpoxyBeads to CD4+ T cells ratios (FACS Aria II, Becton Dickinson).
